# Three-dimensional macroporous graphene monoliths with entrapped MoS_2_ nanoflakes from single-step synthesis for high-performance sodium-ion batteries†

**DOI:** 10.1039/c7ra12617d

**Published:** 2018-01-10

**Authors:** Linfeng Fei, Ming Xu, Juan Jiang, Sheung Mei Ng, Longlong Shu, Li Sun, Keyu Xie, Haitao Huang, Chi Wah Leung, Chee Leung Mak, Yu Wang

**Affiliations:** Department of Applied Physics, The Hong Kong Polytechnic University Hong Kong SAR China apaclmak@polyu.edu.hk; School of Materials Science and Engineering, Nanchang University Nanchang Jiangxi 330031 China wangyu@ncu.edu.cn; School of Metallurgical and Environment, Central South University Changsha 410083 China; Hubei Collaborative Innovation Center for Advanced Organic Chemical Materials, Hubei University Wuhan 430062 China; Beijing Key Laboratory of Materials Utilization of Nonmetallic Minerals and Solid Wastes, National Laboratory of Mineral Materials, School of Materials Science and Technology, China University of Geosciences Beijing 100083 China; State Key Laboratory of Solidification Processing, Center for Nano Energy Materials, Northwestern Polytechnical University and Shaanxi Joint Laboratory of Graphene (NPU) Xi'an 710072 China; Department of Materials Science and NanoEngineering, Rice University Houston Texas 77005 USA

## Abstract

Layered metal sulfides (MoS_2_, WS_2_, SnS_2_, and SnS) offer high potential as advanced anode materials in sodium ion batteries upon integration with highly-conductive graphene materials. However, in addition to being costly and time-consuming, existing strategies for synthesizing sulfides/graphene composites often involve complicated procedures. It is therefore essential to develop a simple yet scalable pathway to construct sulfide/graphene composites for practical applications. Here, we highlight a one-step, template-free, high-throughput “self-bubbling” method for producing MoS_2_/graphene composites, which is suitable for large-scale production of sulfide/graphene composites. The final product featured MoS_2_ nanoflakes distributed in three-dimensional macroporous monolithic graphene. Moreover, this unique MoS_2_/graphene composite achieved remarkable electrochemical performance when being applied to Na-ion battery anodes; namely, excellent cycling stability (474 mA h g^−1^ at 0.1 A g^−1^ after 100 cycles) and high rate capability (406 mA h g^−1^ at 0.25 A g^−1^ and 359 mA h g^−1^ at 0.5 A g^−1^). This self-bubbling approach should be applicable to delivering other graphene-based composites for emerging applications such as energy storage, catalysis, and sensing.

## Abstract 2

A single-step, template-free, high-throughput synthesis method is developed to produce graphene/MoS_2_ composites for improved performances in sodium-ion batteries.

## Graphical abstract

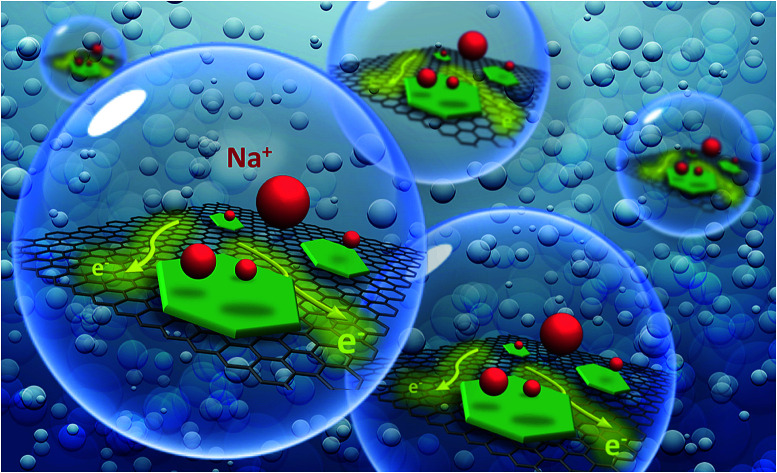

Sodium-ion batteries (NIBs) have been proposed as promising alternatives to lithium-ion batteries (LIBs) in the megawatt- and kilowatt-scale energy storage scenarios (*i.e.*; electric vehicles, stationary grids) for their high cost-effectiveness, sustainability, and environmental benignity.^1^ Since the operation chemistry of NIBs is very similar to that of LIBs, knowledge gained from developing LIB technology can be mostly applied to NIBs with the exception of electrode materials.^2,3^ In particular, the larger ionic radius of Na^+^ (0.102 nm) than that of Li^+^ (0.076 nm) makes graphite, the most commonly used anode in LIBs, unable to accommodate sodium ions in a satisfactory regime.^4^ Inspired by the findings on LIBs, scientists have tested carbonaceous materials,^5,6^ alloy materials (Sn, Sb),^7,8^ and metal oxides (Fe_2_O_3_, CuO, TiO_2_)^9–11^ as anode materials for NIBs. Unfortunately, due to the large volume change and/or the sluggish kinetics during charge/discharge cycles, these materials delivered either low reversible capacity or poor cyclability.^12^ Consequently, layered metal sulfides (MoS_2_, WS_2_, SnS_2_ and SnS) have also been explored as anode materials in NIBs due to their unique structural characteristics.^13^ For example, molybdenum sulfide (MoS_2_), stemming from its large interlayer spacing (0.62 nm, compared to 0.34 nm for graphite) and high capacity for hosting foreign species, has been recently highlighted as a possible candidate for anode material in NIBs.^14–19^ According to the intercalation and conversion reaction between one MoS_2_ molecule and four Na^+^, the theoretical capacity of MoS_2_ is as high as 670 mA h g^−1^.^20^

However, there are two major issues when using MoS_2_ as anodes in large-scale applications: poor electronic conductivity and drastic volume expansion upon conversion reaction from MoS_2_ to Mo and Na_2_S.^18,20–27^ One effective approach to address the problems and thus improve the electrochemical performance of MoS_2_ in NIBs is by supporting MoS_2_ with conductive scaffolds to create porous composites, so as to simultaneously improve its conductivity as well as buffer the volumetric variation.^12^ In this regard, carbon materials, especially graphene, have been repeatedly confirmed to be an efficient conductive additive in electrode materials in resolving the above issues.^28,29^ Some examples of such effective treatment on electrode materials include sulfur/graphene cathode in lithium–sulfur batteries,^30^ lithium metal phosphates/carbon cathode materials in LIBs,^31–34^ and various metal oxides/graphene anode materials in LIBs.^35^

To improve the electrical conductivity and enhance the structural integrity of MoS_2_ anode, MoS_2_/graphene composites have been synthesized *via* several methods and applied in NIBs.^17,18,21,23,26,36–41^ For instance, David *et al.* prepared MoS_2_/graphene composite paper through vacuum filtration of homogeneous dispersions consisting of exfoliated MoS_2_ and graphene oxide sheets, followed by thermal reduction at elevated temperatures.^18^ Wang *et al.* and Xie *et al.* also synthesized MoS_2_/graphene composites *via* hydrothermal reactions plus thermal annealing, respectively.^23,26^ In spite of the significant synthetic achievements made, the existing strategies for synthesizing MoS_2_/graphene composites present a few shortcomings as these methods often involve complicated procedures (graphene oxide preparation, MoS_2_ preparation, compositing or mixing step, thermal treatments, *etc.*) in addition to be costly and time-consuming.^28^ Another issue with existing MoS_2_/graphene compositing methods is that some of them do not ensure the intimate contact between MoS_2_/graphene interfaces, an unfavorable condition for electrochemical applications (charge-transfer process).^26^ Finally, most of the present MoS_2_/graphene compositing methods are faced with the issue of low yield, ranging from several tens to hundreds milligrams of powders under laboratory conditions.

Herein, we report a single-step, template-free, high-throughput “self-bubbling” method for synthesizing MoS_2_/graphene composite. Our method is cost-effective, simple and scalable. The synthesis utilizes the thermal decomposition of solid precursor to generate MoS_2_; meanwhile, the released gas from the decomposition reaction blows premixed, melted glucose into crowded bubbles, which then evolve into graphene structures during annealing. The final product is microscopically featured as highly crystalline MoS_2_ nanoflakes distributed in three-dimensional (3D) macroporous monolithic graphene. With the additional assistance of intimate interfacial contacts between MoS_2_ and graphene, our composite demonstrates considerably improved electrochemical performance when compared with those of conventional MoS_2_/graphene composite upon application in NIBs. It is expected that such a unique MoS_2_/graphene composite should hold potential in promoting the development of practical MoS_2_ anode in NIBs, while the straightforward self-bubbling method could offer the opportunity in producing MoS_2_/graphene composites in industrial scale as well as synthesizing other advanced graphene-based composites.

## Results and discussion

**Fig. 1:**
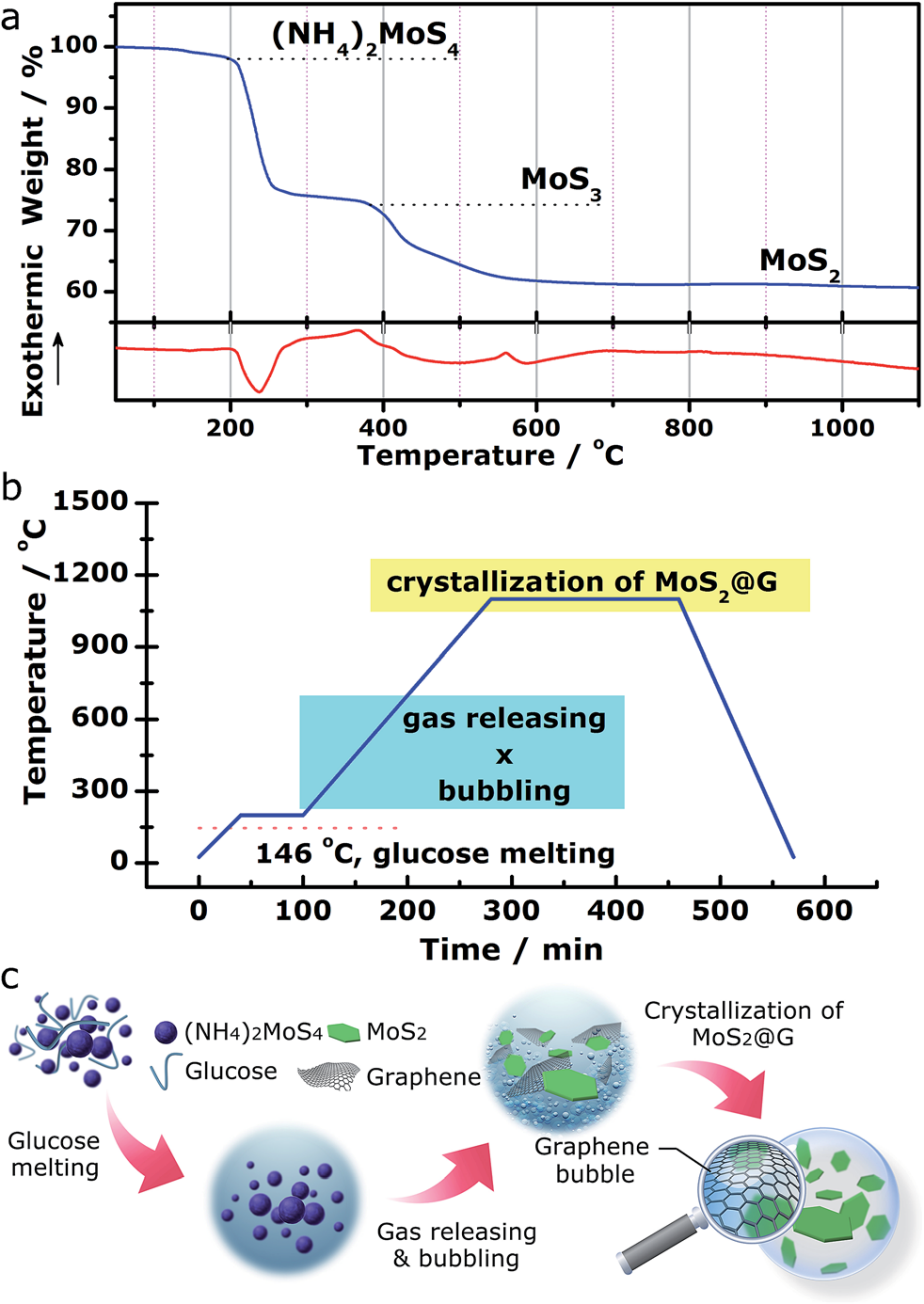
Experimental design of the “self-bubbling” method. (a) TG/DSC profiles of the (NH_4_)_2_MoS_4_ precursor in flowing Ar. (b) The heating program of the “self-bubbling” experiment. (c) The corresponding schematics depicting the synthetic route of the MoS_2_@G hybrid.

We demonstrate a one-step “self-bubbling” system, for the first time, to synthesize the graphene/MoS_2_ composite in this work. Empirically, thermal decomposition of (NH_4_)_2_MoS_4_ in inert atmosphere leads to MoS_2_ while releasing a considerable amount of gases.^42,43^ Results from our carefully conducted thermogravimetric and differential scanning calorimetry (TG/DSC) analysis for (NH_4_)_2_MoS_4_ decomposition in flowing Ar (Fig. 1a) suggests the following processes:

1 (NH_4_)_2_MoS_4_ → 2NH_3_↑ + H_2_S↑ + MoS_3_ (210–380 °C)

2 MoS_3_ → MoS_2_ + S↑ (400–700 °C)

Inspired by these findings, we started with a mixture of (NH_4_)_2_MoS_4_ (as MoS_2_ source) and glucose (as carbon source) with the setup of a two-stage annealing sequence to produce graphene/MoS_2_ hybrid, as shown in Fig. 1b. In the first stage (from room temperature to 200 °C), glucose (m.p. 146 °C) melts into viscous liquid while (NH_4_)_2_MoS_4_ remains solid inside it. Sequentially, during the second stage (from 200 to 1100 °C), (NH_4_)_2_MoS_4_ decomposes into MoS_2_ and crystallizes while the released gas species (ammonia, hydrogen sulfide and sulfur vapor) blow the melted glucose to form crowded bubbles with ultrathin walls, which are then graphitized into 3D graphene networks at high temperature (the released gases actually serve as soft templates to direct the growth of graphene structures.). With this simple approach, a rational nanostructure consists of MoS_2_ and graphene was obtained (hereafter abbreviated as MoS_2_@G hybrid). Besides, our approach also allows the MoS_2_/graphene ratio in the final product to be expediently tuned by using different ratios of (NH_4_)_2_MoS_4_ and glucose as precursors for various potential applications (see Fig. S1,† the composites with varied MoS_2_/graphene ratios). The advantages of such novel approach include low cost, high flexibility, easy operability and excellent scalability. The complete annealing program is presented in Fig. 1b as well as described in the Experimental methods section in the ESI.† A schematic diagram of the whole process is further shown in Fig. 1c.

**Fig. 2:**
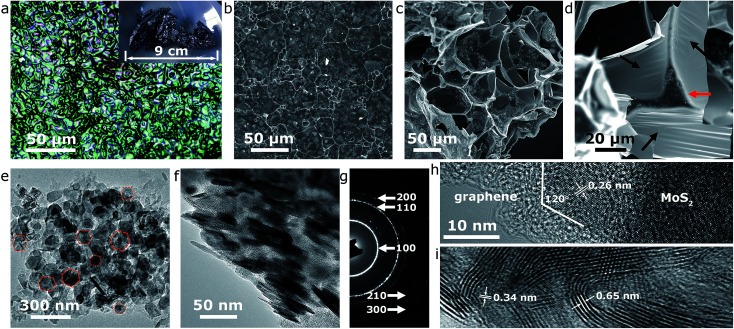
Morphology characterizations of the MoS_2_@G hybrid. (a) Optical image and digital image (inset) of the product. (b–d) SEM images of MoS_2_@G hybrid. (e) Top-view and (f) side-view TEM images of MoS_2_ nanoflakes distributed on few-layered graphene. (g) SAED pattern of MoS_2_@G hybrid. (h and i) HRTEM images of MoS_2_@G hybrid. The scale bar in (h) also applies in (i).

The effectiveness of our approach could be fully confirmed by systematic microstructural analysis of the end-product. Firstly, microscopies were involved to reveal the morphological characteristics of the MoS_2_@G hybrid. The single production of the MoS_2_@G hybrid under our laboratory condition is up to *ca.* 1–2 g when a 1-inch (diameter) tube furnace was used, and the product is foam-like black solid (inset of Fig. 2a). An optical image (Fig. 2a) manifests that the product is composed of large-scale crowded bubbles. The walls of these bubbles are so thin that the light can penetrate through them, leading to rainbow-like reflections on their surface. The scanning electron microscope (SEM) images (Fig. 2b and c) suggest the bubbles are mostly polyhedral units, with a broad size distribution from 1 to 50 micrometers in diameter. Enlarged SEM image (Fig. 2d) further reveals that the wall of the bubbles is made up of ultrathin nanosheets, and every three to four bubbles are interconnected by a strut (denoted by red arrow). One can also notice the presence of large areas of wrinkle-like structures on the nanosheets (denoted by black arrows), a typical phenomenon associated with large-sized graphene, which helps to further increase the surface area of the sample.^44^ Such structure of monolithic graphene is analogous to the 3D Voronoi structure (which is frequently seen in soap bubbles and styrofoam)^45,46^ and provides a number of advantages such as excellent mechanical stability, high surface area, and effective avoidance of the graphene restack.

Our results also show that the graphene walls are decorated with nanosized particulates in the SEM images, most likely the result of the MoS_2_ content. Transmission electron microscope (TEM) images in Fig. 2e and f verify the nanoparticles are quasi-hexagonal nanoflakes (in consistent with our previous *in situ* experiment^22^), with a lateral size of 50–100 nm and thickness of 5–10 nm. As expected, the selected area electron diffraction (SAED) pattern in Fig. 2g can be readily assigned to hexagonal MoS_2_ structure (JCPDS no. 37-1492) while the bright diffraction rings reflect high crystallinity (also refer to Fig. S2,† the energy dispersive spectra (EDS) from the nanoflakes region). The diffraction rings in the SAED pattern correspond to a polycrystalline character, a result of the cumulative signals from many nanoflakes across the selected area aperture (Fig. 2e) although the high-resolution TEM (HRTEM) image in Fig. 2h shows that each MoS_2_ nanoflake is clearly a single crystal. The intimate contact between MoS_2_ nanoflakes and graphene nanosheets is also evident from the TEM images (Fig. 2e–i), a favorable condition for enhancing the electroactivity of the MoS_2_@G hybrid.^26^ Interestingly, despite the validation of the in-plane *d*-spacing of MoS_2_ (0.26 nm for (100) planes) and interlayer distance of graphene (0.34 nm for (002) planes) as shown in the HRTEM images (Fig. 2h and i), a slightly expanded interlayer distance of MoS_2_ (∼0.65 nm, 0.62 nm for natural MoS_2_) can be identified throughout repeated observations. It should be noted that MoS_2_ structure with expanded interlayers is commonly considered to be highly beneficial to improve its electrochemical performance (discharge capacity, reaction kinetics, *etc.*) for battery applications.^20,27^ In short, the above results consistently showed that the sample from our one-step self-bubbling approach was MoS_2_ nanoflakes distributed in macroporous few-layered graphene, in accordance with our original design. For comparison purpose, we also prepared pure MoS_2_ samples *via* the same process without glucose and the final product is mainly irregular microsized flakes (see Fig. S3,† the TEM images of pure MoS_2_ sample).

**Fig. 3:**
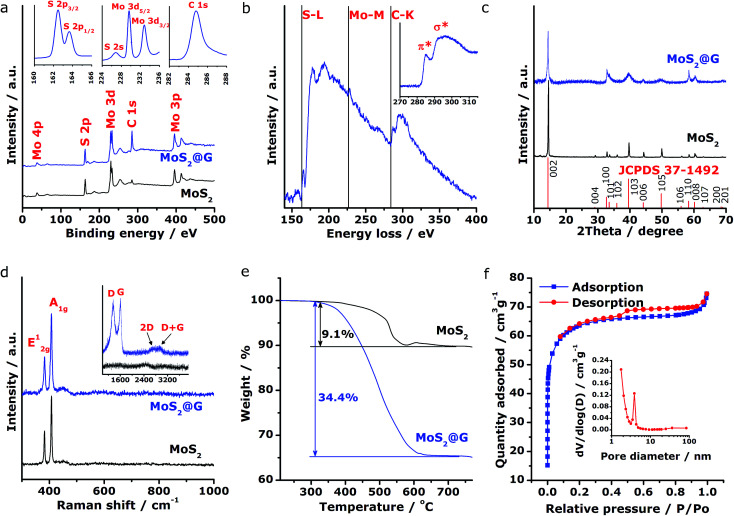
Microstructural analysis of the MoS_2_@G hybrid and the MoS_2_. (a) XPS spectra of MoS_2_@G hybrid and MoS_2_, with high-resolution S 2p, Mo 3d, and C 1s spectra for the MoS_2_@G hybrid as the insets. (b) EELS spectrum and the core-loss C K-edge spectrum (inset) of MoS_2_@G hybrid. (c) Comparison of the XRD patterns of MoS_2_@G hybrid and MoS_2_. (d) Raman spectra of MoS_2_@G hybrid and MoS_2_ in MoS_2_ region and graphene region (inset). (e) Thermogravimetric profiles for MoS_2_@G hybrid and MoS_2_, obtained by annealing the samples in synthetic air. (f) N_2_ adsorption/desorption isotherms of MoS_2_@G hybrid and the corresponding pore-size distribution (inset).

Subsequently, spectroscopic characterizations were employed to further explore the microstructural features of the MoS_2_@G hybrid. Concerning the chemical states of Mo and S in the product, Fig. 3a shows the X-ray photoelectron spectroscopy (XPS) survey scans for MoS_2_ and MoS_2_@G hybrid, with their C 1s peak referenced at 284.8 eV. The presence of MoS_2_ with Mo and S elemental ratio of ∼1 : 2 can be identified for both samples, besides the prominent carbon component in the MoS_2_@G hybrid. The insets in Fig. 3a show the high-resolution spectra of MoS_2_@G hybrid, which are the S 2p, Mo 3d and C 1s regions. The Mo 3d possesses two peaks centered at 229.6 and 232.8 eV, in association with the doublet Mo 3d_5/2_ and Mo 3d_3/2_ for Mo^4+^ ions. Another group of peaks, ascribed to the S 2p_3/2_ and S 2p_1/2_ orbital of divalent sulfide ions (S^2−^), are observed at 162.4 and 163.7 eV, respectively. All these results are well consistent with the reported values for MoS_2_.^25,47^ The existence of Mo, S and C in the MoS_2_@G hybrid was also verified by the electron energy-loss spectrum (EELS), as shown in Fig. 3b by the S L-edge, Mo M-edge and C K-edge. Particularly, the core-loss C K-edge EELS spectrum of the MoS_2_@G hybrid (inset in Fig. 3b) presents a sharp π* peak (∼284 eV, due to the excitation from 1s spin level to empty π* orbits of the sp^2^-bonded atoms) as well as a clear σ* step (∼289 eV, resulting from the transition from the 1s level to empty σ* orbits at both sp^2^ and sp^3^-bonded atoms), suggesting the high crystalline nature of graphene in the hybrid.^30,48^

The crystal structure of the samples was then studied by X-ray diffraction (XRD). As shown in Fig. 3c, the XRD patterns for both MoS_2_ and MoS_2_@G hybrid match with 2H molybdenite; however, the diffraction peaks of the MoS_2_@G hybrid are much boarder than those of pure MoS_2_, a result of the fine MoS_2_ crystalline size. Notably, the peak of (002) planes for the MoS_2_@G hybrid slightly shifts towards the direction of low scattering angle, corroborating the expanded interlayer distance as revealed by the above TEM results. The diffraction signal for graphene is not visible due to the intense peaks from MoS_2_ crystals, so Raman measurement was applied. As can be seen from the inset of Fig. 3d, the Raman spectrum for MoS_2_@G hybrid exhibits two sharp bands at 1360 (D band, in-plane vibration of sp^3^-bonded carbon) and 1600 cm^−1^ (G band, vibration mode of sp^2^-bonded carbon), as well as two broad bands at 2690 (2D band) and 2920 cm^−1^ (D + G band),^49^ which directly proved the existence of well-crystallized few-layered graphene structure in the hybrid. The hexagonal layered structure of the MoS_2_ in the hybrid was further confirmed by Raman spectrum with two peaks located at 383 and 407 cm^−1^ (Fig. 3d), which are typical E^1^_2g_ and A_1g_ modes due to in-plane vibrations within the sulfur–molybdenum–sulfur layers, respectively.^50^ Consequently, the carbon content in the MoS_2_@G hybrid was measured by annealing the sample in synthetic air upon TG/DSC test. Assuming the complete formation of MoO_3_, SO_2_ and CO_2_,^51^ the graphene content is estimated to be 27.8 wt%, corresponding to the MoS_2_ content of 72.2 wt% (Fig. 3e).

To further characterize the composite structure, the specific surface area and porous nature of the MoS_2_@G hybrid was quantified by Brunauer–Emmett–Teller (BET) method. Results from the full nitrogen adsorption and desorption isotherms (Fig. 3f) present typical type-IV characteristics with type-H4 hysteresis loop at a relative pressure above 0.5, indicating a nanoporous structure. Accordingly, the surface area of the MoS_2_@G hybrid is as high as 196.93 m^2^ g^−1^; in contrast, the surface area of the MoS_2_ sample is 6.22 m^2^ g^−1^ (Fig. S4†). It is also worth noting that the pore sizes of the MoS_2_@G hybrid, derived *via* the Barrett–Joyner–Halenda (BJH) method, are mainly distributed in the region of mesopores to macropores with a peak centered at 3.87 nm (inset in Fig. 3f). The high surface area of the MoS_2_@G hybrid together with the ample pores would be extremely favorable for energy storage applications such as batteries. The porosity in the Voronoi-structured framework can act as efficient electrolyte reservoirs to enlarge the contact areas between electrolyte and the active materials, and increase the active sites for sodiation/desodiation. Meanwhile, the porosity can also buffer the volume change to avoid structural pulverization during repeated charge/discharge cycles.

**Fig. 4:**
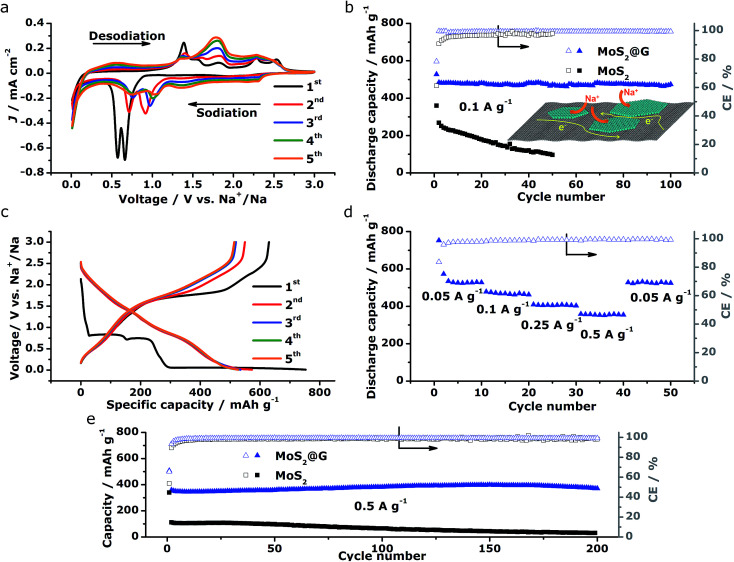
Electrochemical performances of the MoS_2_@G hybrid and the MoS_2_ as anode materials in NIBs. (a) The initial CV profiles of MoS_2_@G hybrid measured at 0.2 mV s^−1^ in the voltage window of 0.01–3 V. (b) Comparison of cycling performances of MoS_2_@G hybrid and MoS_2_ at 0.1 A g^−1^ and the schematic of transport paths for Na^+^ and electrons in the MoS_2_@G hybrid (inset). (c) The initial galvanostatic discharge/charge curves for MoS_2_@G hybrid at 0.05 A g^−1^ in the voltage range of 0.01–3 V. (d) Rate performance of MoS_2_@G hybrid at programmed current densities. (e) Comparison of the long-term cycling behaviors of MoS_2_@G hybrid and MoS_2_ at 0.5 A g^−1^.

The above structural characterizations of our MoS_2_@G hybrid suggest the high potential of applying the product as electrode materials in NIBs. To verify this, systematic electrochemical measurements were performed with CR2032 coin cells. Fig. 4a shows the cyclic voltammograms (CVs) of MoS_2_@G hybrid during the initial five cycles in the potential range of 0.01–3 V *versus* Na^+^/Na. In the first cathodic scan (sodiation), the first peak at 1.20 V can be ascribed to the intercalation of sodium ions into MoS_2_ interlayer (refer to Fig. S5† for the isolated first scan).^24^ The following two reduction peaks at 0.66 and 0.56 V are attributed to the two-step insertion of Na^+^ into MoS_2_.^26,52^ The fourth subtle peak located at ∼0.35 V is related to the conversion reaction from MoS_2_ to Mo and Na_2_S.^18^ The last sharp cathodic peak at 0.02 V is associated with the intercalation of Na^+^ into the graphene interlayers.^53^ In the subsequent anodic scan (desodiation), the peaks from 1.4 to 1.7 V should be attributed to the oxidation of Mo to MoS_2_.^27^ In the subsequent cycles, the peaks at 0.66/0.56 V shift to 1.04/0.75 V with decreased intensity, corresponding to the progressive amorphization of MoS_2_@G hybrid. Notably, the CVs rapidly become overlapped in the later cycles, suggesting high reversibility for the electrode material. The stability of sodiation/desodiation processes was also confirmed by comparing the cycling performances of MoS_2_@G hybrid and MoS_2_ under the galvanostatic mode at a current density of 0.1 A g^−1^, as shown in Fig. 4b. First, both samples show initial capacity drops as well as low coulombic efficiencies (CE) at the first cycle, which should be a result of the formation of solid electrolyte interface (SEI) film. Second, the MoS_2_@G hybrid delivered a capacity as high as 484 mA h g^−1^ at the 2nd cycle and 474 mA h g^−1^ at the 100th cycle (corresponding to a small capacity decay of 0.02% per cycle). In contrast, the MoS_2_ electrode delivered only a capacity of 268 mA h g^−1^ at the 2nd cycle and 97 mA h g^−1^ at the 50th cycle. Furthermore, the CE of the MoS_2_@G hybrid (>99%) is constantly higher than that of MoS_2_.

**Fig. 5:**
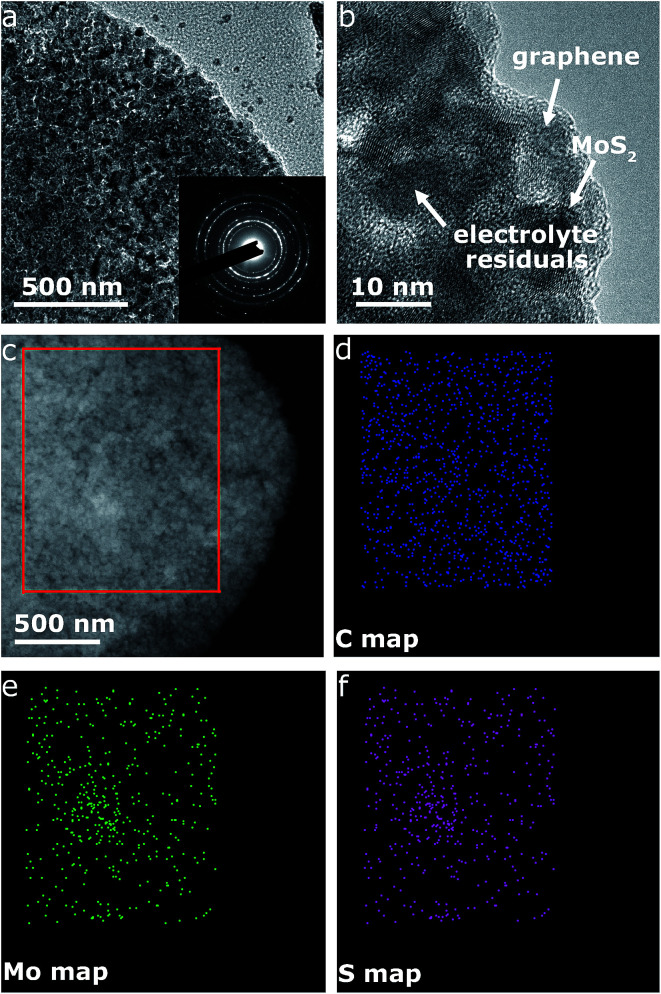
TEM characterizations for MoS_2_@G hybrid after cycling. (a) TEM image and the corresponding SAED pattern (inset); (b) magnified high-resolution TEM image (the discharged products and electrolyte residuals are visible as irregular particles); (c) the STEM (scanning transmission electron microscopy) image of the MoS_2_@G hybrid and the corresponding (d) C, (e) Mo, and (f) S maps from the region indicated by the red rectangle in (c).

Fig. 4c displays the galvanostatic discharge/charge voltage profiles during the first five cycles of the MoS_2_@G hybrid at 0.05 A g^−1^ in the potential window of 0.01–3 V *vs.* Na^+^/Na. The distinct discharge/charge plateaus in the first cycle are ascribed to the sample's high crystallinity.^23^ The discharge and charge capacities for the first cycle are 750 and 630 mA h g^−1^, respectively, corresponding to a CE of 83.7% (in line with the low initial CE in Fig. 4b). In the subsequent cycles, the discharge and charge profiles become identical, and no obvious discharge/charge plateau can be identified. These cells were then involved in the test of rate capability and the result is presented in Fig. 4d. The specific discharge capacities are 530, 475, 408, and 357 mA h g^−1^ at 0.05, 0.1, 0.25, and 0.5 A g^−1^, respectively; *i.e.*, when the current density is increased by ten times (from 0.05 to 0.5 A g^−1^), the electrode material can still retain ∼67% of its capacity (from 530 to 357 mA h g^−1^). Moreover, the MoS_2_@G hybrid is able to recover most of its original capacity when the current rate is restored back to 0.05 A g^−1^ after forty deep cycles, indicating the high stability of the MoS_2_@G anode even upon high rate cycling (also refer to Fig. S6–S8† for the CV profiles, discharge/charge curves, and rate performance of the MoS_2_ sample, as well as Fig. S9† for the cycling performance of graphene). Therefore, another cycling test was conducted under the galvanostatic mode at a higher current density (0.5 A g^−1^, Fig. 4e). After 200 cycles, the MoS_2_@G anode remarkably preserves its sodium storage capacity at as high as 371 mA h g^−1^ whereas the MoS_2_ anode almost loses its electrochemical activity (final capacity of 31 mA h g^−1^). It is worth noting that, during these repeated discharge/charge cycles, the volume change induced pulverization of the MoS_2_@G hybrid electrode has been significantly suppressed, as reflected by the constantly high CE (>99%) throughout the measurement and the comparative postmortem TEM study. As shown in Fig. 5, the postmortem TEM study of MoS_2_@G hybrid indicates the existence of small MoS_2_ flakes firmly decorated on graphene sheets. The MoS_2_ content remains highly crystallized except the irregular outlines after such long cycling. Moreover, the uniform distribution of MoS_2_ on graphene sheets was also successful maintained. In contrast, the MoS_2_ sample shows considerable cracks across the flakes throughout the cycling (Fig. S10†).

The above electrochemical characterizations substantiate the fact that our MoS_2_@G hybrid produced by the novel “self-bubbling” approach possesses high reversible capacity, excellent rate capability, as well as superior cycling stability as anode material in NIBs. Such impressive performance, to the best of our knowledge, is among one of the best values for MoS_2_-based anode materials for NIBs (refer to Table S1,† the comparison of electrochemical performances with selected MoS_2_-based anode materials for NIBs). We believe that the unique microstructural features of the MoS_2_@G hybrid itself have brought multiple advantages to act as high-performance electrode materials. First, the thin MoS_2_ nanoflakes (5–10 nm in thickness) with expanded interlayer spacing (0.65 nm) greatly reduces the strain caused by insertion and extraction of Na^+^. Second, the high surface area of the MoS_2_@G hybrid (196.93 m^2^ g^−1^) provides a large electrode–electrolyte interface, in facilitating the critical charge-transfer process. Third, the monolithic graphene ensures a 3D porous and flexible scaffold to protect MoS_2_ from dissolution or detachment during repeated cycles, as well as 3D interconnected pathways for electron transport and Na-ion diffusion. Finally, the homogeneous distribution of MoS_2_ nanoflakes on graphene nanosheets together with their intimate contact guarantees good conductivity (for both Na^+^ and electrons), and hence a high level of electrochemical activity and effective material utilization of the MoS_2_ (as shown in the inset of Fig. 4b).

## Conclusions

In summary, we have developed a simple and scalable “self-bubbling” approach for synthesizing an advanced MoS_2_@G hybrid composed of MoS_2_ nanoflakes entrapped in 3D macroporous monolithic graphene frameworks. Benefiting from the unique microstructural characteristics, the MoS_2_@G hybrid has shown outstanding electrochemical performance including remarkable cycling stability and high rate capability upon used as anode material for NIBs. On account of the promising structural tunability of the MoS_2_@G hybrid, we also believe that the product might possess great potential in other application areas such as supercapacitors, catalysts, and sensors. Our one-step method may be also applicable in constructing other emerging graphene-based composites, and should therefore inspire further attempts to additional application scenes in future.

## Experimental methods

### Synthesis of MoS_2_@G hybrid

The MoS_2_@G hybrid was synthesized from a novel “self-bubbling” approach. A mixed powder of (NH_4_)_2_MoS_4_ and glucose (weight ratio of 1 : 1.5) was directly subjected to thermal treatment in a horizontal furnace. As shown in Fig. 1b, the temperature was first ramped from room temperature to 200 °C in 40 minutes and this temperature was kept for 60 minutes. The temperature was then further increased to 1100 °C in 180 minutes and hold for another 180 minutes. The product was harvested after cooling the system to room temperature in 110 minutes. The whole annealing process was protected by a constant argon flow.

### Material characterizations

The samples were characterized by different analytical techniques. Simultaneous TG/DSC analysis was performed on a NETZSCH STA 449 C Jupiter system. Optical image was captured on a Nikon Microphot-FXA microscope. SEM observations were made on a JEOL JSM-6700F field-emission SEM. TEM images, SAED pattern, EELS, and EDS were obtained on a JEOL JEM-2100F STEM (200 kV, field-emission gun) system equipped with an Oxford INCA x-sight EDS and an ENFINA 1000 EELS. XPS spectra were acquired on a Thermo Scientific Escalab 250Xi spectrometer. XRD measurement was conducted using a Rigaku SmartLab Intelligent X-ray diffraction system with filtered Cu K_α_ radiation (*λ* = 1.5406 Å, operating at 45 kV and 200 mA). Raman measurement was taken using a Horiba Jobin Yvon LabRAM HR system with a laser wavelength of 488 nm. The nitrogen adsorption and desorption isotherms were obtained at 77 K with a Micromeritics ASAP 2020 volumetric adsorption analyzer.

### Electrochemical measurements

The working electrode slurry was prepared by mixing the active materials with Super P and carboxymethyl cellulose binder at a mass ratio of 8 : 1 : 1. The slurry was then spread on the surface of a copper foil and dried at 60 °C for 12 h. Finally, the electrode was stamped into disks with a diameter of 10 mm and vacuum-dried at 60 °C for another 6 h. With sodium tablets as the reference electrode and glass fiber membrane as the separator, CR2032 coin cells were assembled in a glove box (MIKROUNA-Universal-2440-1750) filled with argon. 1 M NaClO_4_ in the mixed solvent of ethylene carbonate/dimethyl carbonate (1 : 1 v/v ratio) with 5 wt% fluoroethylene carbonate as additive was selected as the electrolyte for the coin cells. CV measurement was conducted on an electrochemical measurement system (PARSTAT 2273) with a scan rate of 0.2 mV s^−1^ from 3 to 0.01 V. Galvanostatic charge/discharge tests were performed by using a battery testing system (LAND CT2001A) within the potential of 0.01–3 V at room temperature. The capacities are given with respect to the total mass of the active materials throughout the work.

## Author contributions

L. F., C. L. M., and Y. W. conceived the idea and designed the experiment. L. F. and J. J. prepared samples. L. F., J. J., S. M. N., L.-L. S., L. S., and K. X. conducted structural characterizations. L. F. and M. X. performed electrochemical measurements. H. H. and C. W. L. contributed to critical discussions. L. F., C. L. M., and Y. W. wrote the manuscript, and all authors commented on it. Y. W. and C. L. M. supervised implementation of project. All authors have given approval to the final version of the manuscript. L. F. and M. X. contributed equally to this work.

## Conflicts of interest

There are no conflicts to declare.

## Supplementary Material

RA-008-C7RA12617D-s001
