# Supplementary material for: Three-dimensional macroporous graphene monoliths with entrapped MoS2 nanoflakes from single-step synthesis for high-performance sodium-ion batteries
Source: RSC Adv. 2018 Jan 10;8(5):2477–84. doi: 10.1039/c7ra12617d (PMC9077459; doi:10.1039/c7ra12617d)
Supplement: RA-008-C7RA12617D-s001 [file RA-008-C7RA12617D-s001.pdf]

Electronic Supplementary Material (ESI) for RSC Advances.
This journal is © The Royal Society of Chemistry 2018

Three-dimensional macroporous graphene monoliths with entrapped MoS_2 nanoflakes from single-step synthesis for high-performance sodium-ion batteries

Linfeng Fei, Ming Xu, Juan Jiang, Sheung Mei Ng, Longlong Shu, Li Sun, Keyu Xie, Haitao Huang, Chi Wah Leung, Chee Leung Mak, and Yu Wang**

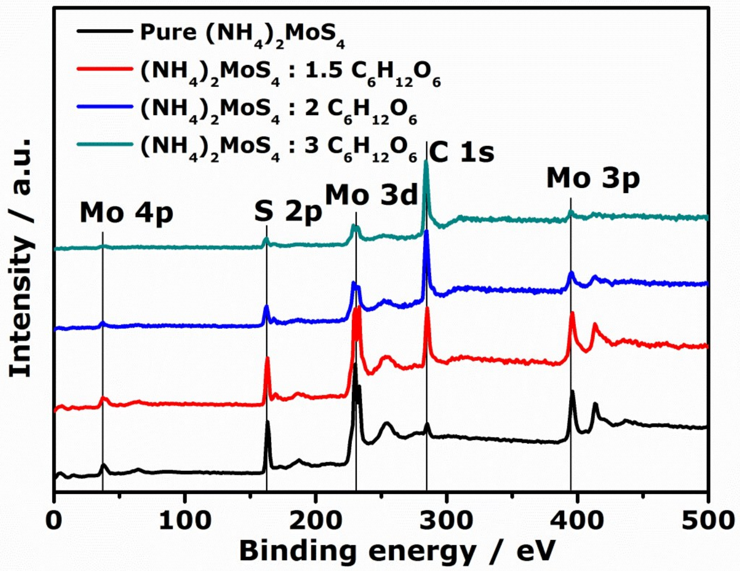

Figure S1. XPS survey profiles for different MoS_2@G hybrid composites from varied ratios of $(NH_4)_2MoS_4$ and glucose as precursors. From XPS measurement, the weight percentages of carbon in the composites can be estimated as 3.1% (black line), 28.1% (red line), 37.3% (blue line), and 52.7% (cyan line).

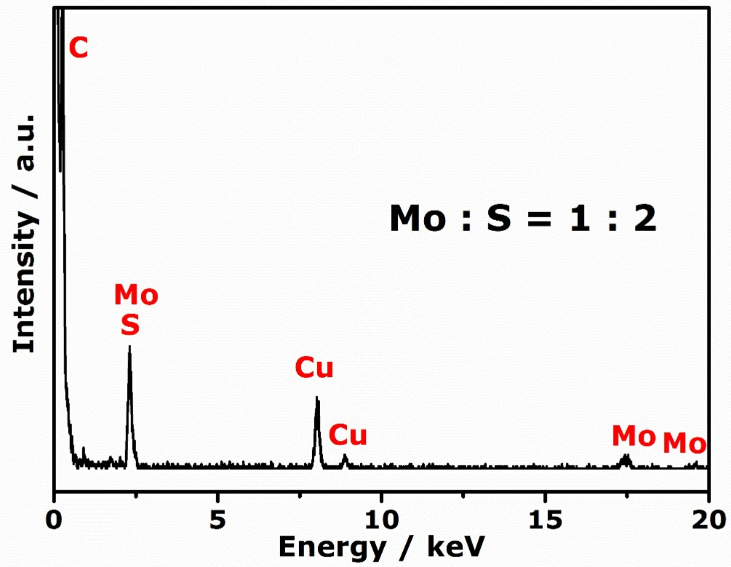

Figure S2. The EDS spectra of the MoS_2@G hybrid from the nanoflakes region, showing the atomic ratio for Mo and S is roughly 1:2.

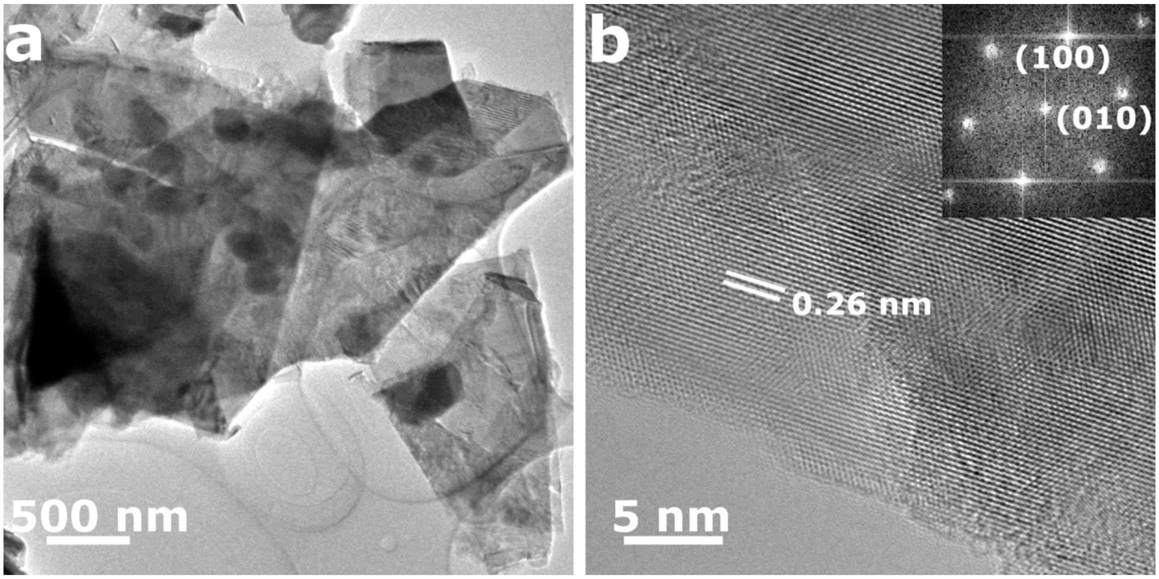

Figure S3. (a) The TEM and (b) HRTEM images for the pure MoS_2 sample. The inset in (b) is the SAED pattern from an isolated flake.

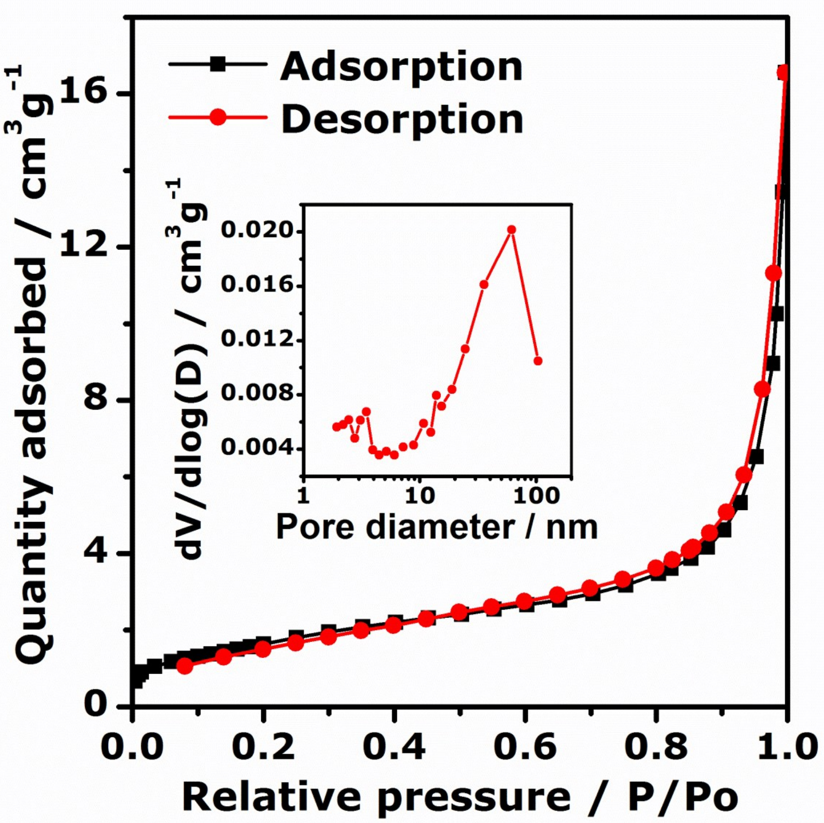

Figure S4. N_2 adsorption/desorption isotherms for the MoS_2 sample and the corresponding pore-size distribution (inset).

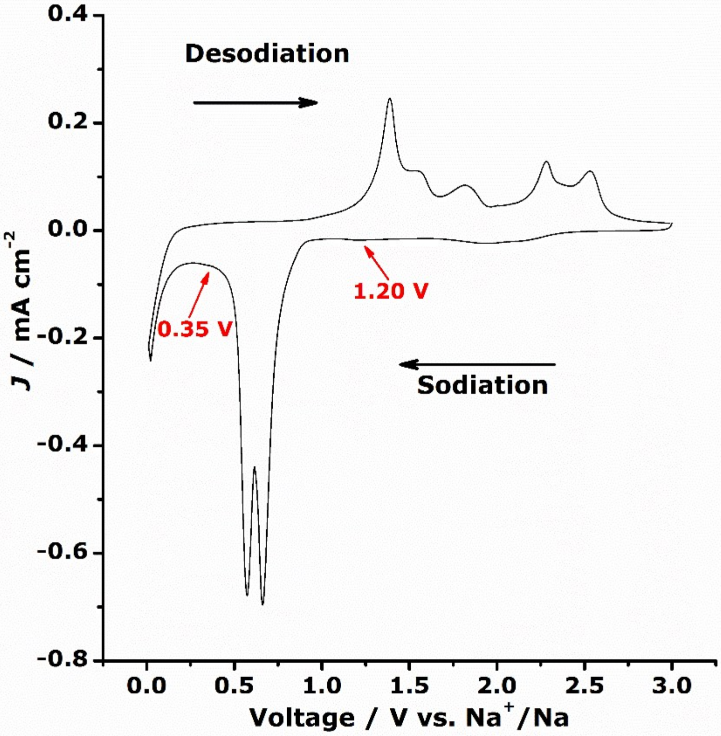

Figure S5. The initial CV profiles of MoS_2@G hybrid measured at 0.2 mV s^{-1} in the voltage window of 0.01 - 3 V.

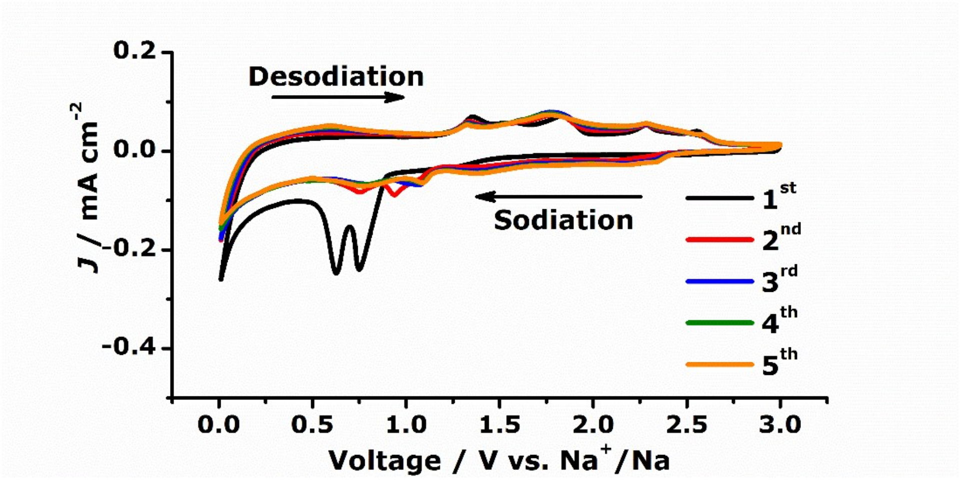

Figure S6. The initial CV profiles of MoS_2 sample measured at 0.2 mV s^{-1} in the voltage window of 0.01 - 3 V.

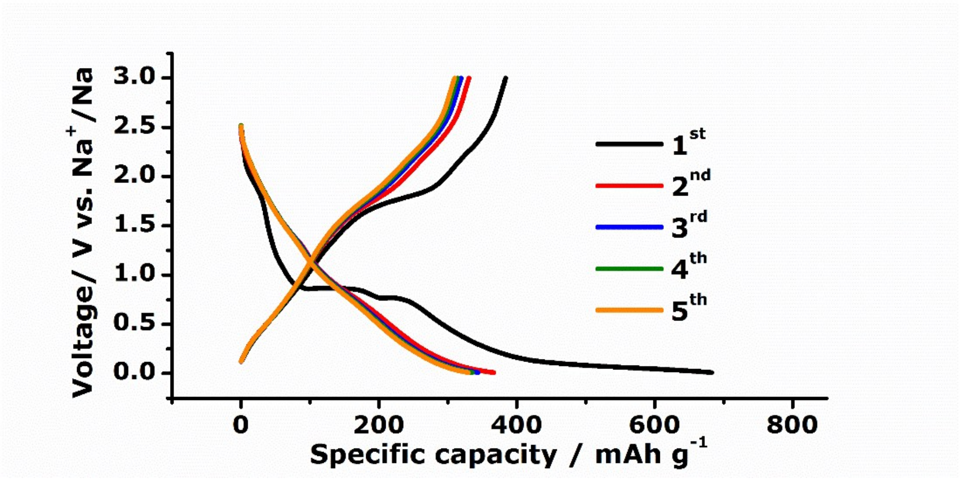

Figure S7. The initial galvanostatic discharge/charge curves for MoS_2 sample at 0.05 A g^{-1} in the voltage range of 0.01 - 3 V.

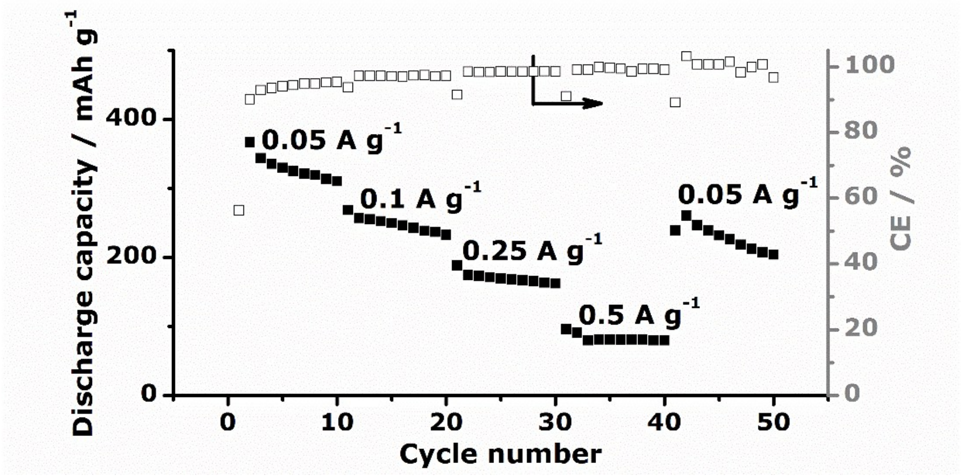

Figure S8. Rate performance of MoS_2 sample at programmed current densities.

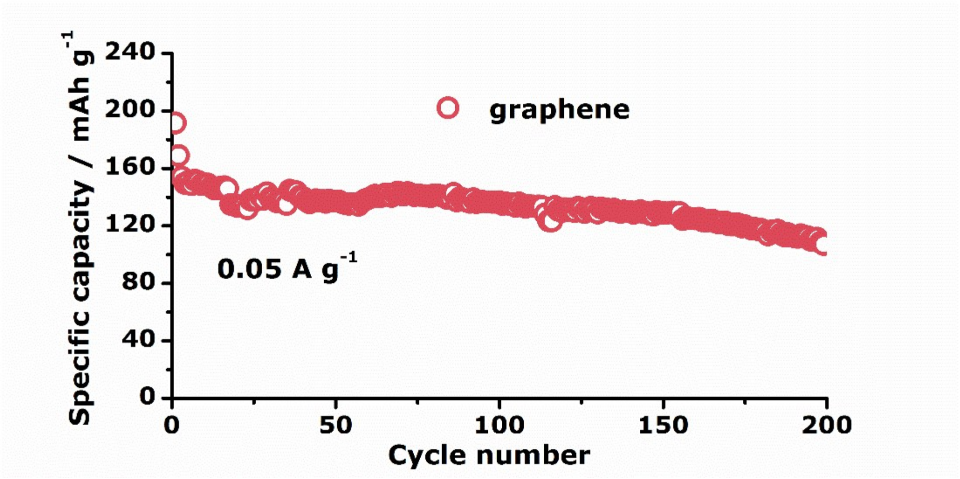

Figure S9. The cycling performance of graphene measured at 0.05 A g^{-1}.

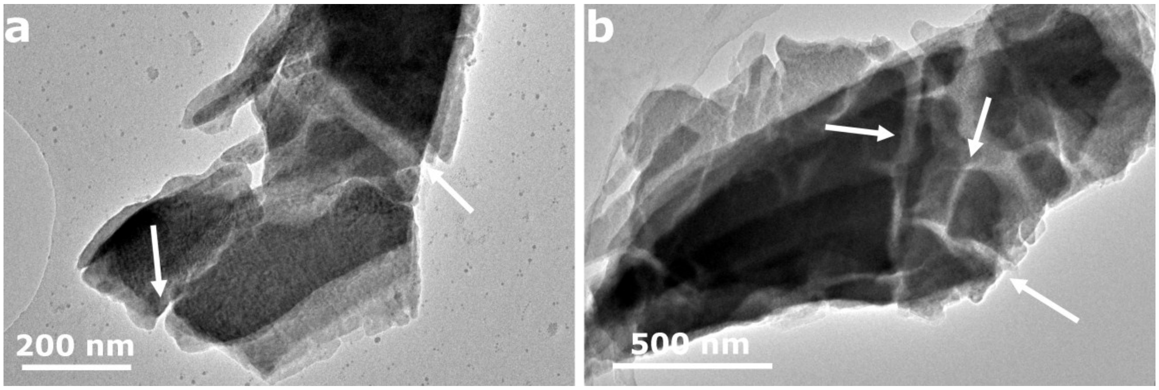

Figure S10. TEM characterizations of the MoS_2 flakes after cycling. The white arrows in both panels indicate the pulverization of flakes after repeated sodiation/desodiation.

Table S1. Comparison of electrochemical performances for the reported MoS_2-based materials (calculated on the whole composite).

MoS_2-based materials	Cyclability (capacity retention, compared with the 2nd cycle)	Rate performance	Ref.
**MoS_2@G hybrid**	484 mAh g^{-1} at 0.1 A g^{-1}, 100 cycles (98%) 371 mAh g^{-1} at 0.5 A g^{-1}, 200 cycles	357 mAh g^{-1} at 0.5 A g^{-1}	This work
**MoS_2/amorphous carbon microspheres**	425 mAh g^{-1} at 0.3 A g^{-1}, 100 cycles (94%)	186 mAh g^{-1} at 7.0 A g^{-1}	[1]
**MoS_2/C nanospheres**	381 mAh g^{-1} at 0.067 A g^{-1}, 50 cycles (77%)	294 mAh g^{-1} at 1.34 A g^{-1}	[2]
**MoS_2/Graphene Microspheres**	480 mAh g^{-1} at 0.2 A g^{-1}, 50 cycles (88%)	234 mAh g^{-1} at 10.0 A g^{-1}	[3]
**Single-layerd MoS_2/carbon nanowire**	436 mAh g^{-1} at 1.0 A g^{-1}, 100 cycles (60%)	139 mAh g^{-1} at 20.0 A g^{-1}	[4]
**MoS_2/graphene composite**	218mAh g^{-1} at 0.025 A g^{-1}, 20 cycles (83%)	173 mAh g^{-1} at 0.2 A g^{-1}	[5]
**MoS_2/graphene**	313 mAh g^{-1} at 0.1 A g^{-1}, 200 cycles (81%)	280 mAh g^{-1} at 0.1 A g^{-1}	[6]
**Ultrathin MoS_2 nanosheets**	386 mAh g^{-1} at 0.04 A g^{-1}, 100 cycles (73%)	251 mAh g^{-1} at 0.32 A g^{-1}	[7]
**Mesoporous MoS_2/C microspheres**	484 mAh g^{-1} at 0.1 A g^{-1}, 100 cycles (94%)	244 mAh g^{-1} at 20.0 A g^{-1}	[8]

References

(1) Choi, S. H.; Kang, Y. C. Polystyrene-Templated Aerosol Synthesis of MoS_2-Amorphous Carbon Composite with Open Macropores as Battery Electrode. *ChemSusChem* **2015**, *8*, 2260–2267.

(2) Wang, J.; Luo, C.; Gao, T.; Langrock, A.; Mignerey, A. C.; Wang, C. An Advanced MoS_2/Carbon Anode for High-Performance Sodium-Ion Batteries. *Small* **2015**, *11*, 473–481.

(3) Choi, S. H.; Ko, Y. N.; Lee, J.-K.; Kang, Y. C. 3D MoS_2-Graphene Microspheres Consisting of Multiple Nanospheres with Superior Sodium Ion Storage Properties. *Adv. Funct. Mater.* **2015**, *25*, 1780–1788.

(4) Zhu, C.; Mu, X.; van Aken, P. A.; Yu, Y.; Maier, J. Single-Layered Ultrasmall Nanoplates of MoS_2 Embedded in Carbon Nanofibers with Excellent Electrochemical Performance for Lithium and Sodium Storage. *Angew. Chem. Int. Ed.* **2014**, *53*, 2152–2156.

(5) David, L.; Bhandavat, R.; Singh, G. MoS_2/Graphene Composite Paper for Sodium-Ion Battery Electrodes. *ACS Nano* **2014**, *8*, 1759–1770.

(6) Wang, Y.-X.; Chou, S.-L.; Wexler, D.; Liu, H.-K.; Dou, S.-X. High-Performance Sodium-Ion Batteries and Sodium-Ion Pseudocapacitors Based on MoS_2/Graphene Composites. *Chem. - A Eur. J.* **2014**, *20*, 9607–9612.

(7) Su, D.; Dou, S.; Wang, G. Ultrathin MoS_2 Nanosheets as Anode Materials for Sodium-Ion Batteries with Superior Performance. *Adv. Energy Mater.* **2015**, *5*, 1401205.

(8) Lu, Y.; Zhao, Q.; Zhang, N.; Lei, K.; Li, F.; Chen, J. Facile Spraying Synthesis and High-Performance Sodium Storage of Mesoporous MoS_2/C Microspheres. *Adv. Funct. Mater.* **2016**, *26*, 911–918.
